# Mepolizumab efficacy in chronic obstructive pulmonary disease: insights from longitudinal patterns of blood eosinophil counts and their variability across 3 clinical trials

**DOI:** 10.1093/ajrccm/aamag288

**Published:** 2026-06-09

**Authors:** Gerard J Criner, Henrik Watz, MeiLan K Han, Fernando J Martinez, Steven Gould, Jeff Min, Arunangshu Biswas, Stefanie Kolterer, Dave Singh

**Affiliations:** Department of Thoracic Medicine and Surgery, Lewis Katz School of Medicine, Temple University, Philadelphia, PA, United States; Velocity Clinical Research Germany GmbH, Ahrensburg, Germany; Medical Clinic III, Campus Lübeck, University Hospital Schleswig-Holstein, Airway Research Center North, German Center For Lung Research (DZL), Lübeck, Germany; Pulmonary and Critical Care, University of Michigan Health System, Ann Arbor, MI, United States; Division of Pulmonary, Allergy, and Critical Care Medicine, UMass Memorial Health, University of Massachusetts Chan Medical School, Worcester, MA, United States; Global Medical Affairs, Specialty Care, GSK, London, United Kingdom; Clinical Research–Respiratory, GSK, Waltham, MA, United States; Biostatistics, GSK, Bengaluru, Karnataka, India; Global Medical Affairs, Specialty Care, GSK, London, United Kingdom; Division of Immunology, Immunity to Infection and Respiratory Medicine, School of Biological Sciences, The University of Manchester, Manchester University NHS Foundation Trust, Manchester, United Kingdom; Medicines Evaluation Unit, an IQVIA business, Manchester, United Kingdom

**Keywords:** biological products, pulmonary disease, chronic obstructive, IL-5, eosinophils

## Abstract

**Rationale:**

Blood eosinophil count (BEC) is a biomarker indicating type 2 inflammation in patients with chronic obstructive pulmonary disease (COPD), but can be variable.

**Objectives:**

Evaluate mepolizumab efficacy in patients with COPD with variability of BEC patterns.

**Methods:**

Data were pooled from the phase 3, randomized, double-blind, placebo-controlled METREX (NCT02105948), METREO (NCT02105961), and MATINEE (NCT04133909) trials. Patients receiving mepolizumab 100 mg or placebo were included. These trials documented historical BEC (within 12 months before randomization) for all patients. Outcomes included annualized rates of moderate or severe exacerbations, and exacerbations requiring an emergency department (ED) visit and/or hospitalization. Outcomes were assessed in subgroups based on BEC values in the 12 months before randomization, at screening, and at baseline.

**Measurements and Main Results:**

Annualized rates of moderate or severe exacerbations were reduced, or trended toward reduction, with mepolizumab versus placebo in patients with type 2 inflammation characteristics, including those with BEC ≥300 cells/µL at any pre-randomization timepoint (21% reduction), persistently elevated BECs (12%-27% reduction), and ­variable BECs over time (22%-36% reduction). Across various subgroups with elevated BEC, starting from ≥150 cells/µL, mepolizumab reduced exacerbations requiring ED visit and/or hospitalization. Patients with persistently low BEC (<150 cells/µL) experienced no benefit with mepolizumab.

**Conclusions:**

Mepolizumab improved exacerbation-related outcomes in patients with COPD and type 2 inflammation, characterized by both consistently and intermittently elevated BEC. Persistent BEC elevation is not required to identify treatable eosinophilic phenotypes in COPD.

At a Glance Commentary **Current Scientific Knowledge on the Subject:** Blood eosinophil counts (BECs) provide biomarker information regarding the presence of type 2 inflammation in chronic obstructive pulmonary disease (COPD), and are used to identify patients for pharmacological interventions targeting this pathway, such as biologics. BECs fluctuate, and, in real-world datasets, patients with persistently high BECs are uncommon, with most being considered as intermittently eosinophilic. It is important to understand how BEC variability impacts treatment response to biologic therapies targeting type 2 inflammation in COPD.

**What This Study Adds to the Field:** This analysis shows consistent reduction of exacerbations by mepolizumab among patients with COPD and an eosinophilic phenotype, both in those with persistently elevated BECs and in those with variability in their BECs. These data provide guidance on eosinophil testing to inform therapeutic decisions, by showing the efficacy of mepolizumab versus placebo whether a high BEC is persistent or intermittent, and irrespective of the timing of measurement before treatment implementation.

## Introduction

Blood eosinophil counts (BECs) provide biomarker information regarding the presence of type 2 inflammation in chronic obstructive pulmonary disease (COPD), as well as other diseases, and are used to identify patients for pharmacological interventions targeting this pathway, such as biologics.^1^^,^^2^ Elevated BECs are associated with an increased risk of COPD exacerbations and accelerated lung function decline.^3-8^ Threshold values to identify individuals with a type 2 inflammatory component in various diseases range from BEC ≥150 cells/µL to ≥300 cells/µL, while cutoffs between <100 cells/µL and <150 cells/µL have been used to identify individuals without type 2 inflammation.^2^^,^^9-12^

BECs fluctuate and depend on intrinsic and extrinsic factors,^1^^,^^6^^,^^9^^,^^13^^,^^14^ such as circadian rhythm and medication use (including oral corticosteroids for exacerbations, and inhaled corticosteroids [ICS] for maintenance treatment).^14-18^ While BECs that remain consistently <150 cells/µL are indicative of an absence of type 2 inflammation, the presence of high levels of type 2 inflammation (BEC ≥300 cells/µL) often varies within the same individual^14^^,^^19-21^; in real-world datasets, patients with persistently high BECs ≥300 cells/µL are uncommon, with most being considered as intermittently eosinophilic.^14^^,^^19^^,^^20^ It is important to understand how BEC variability impacts treatment response to biologic therapies targeting type 2 inflammation in COPD.

Interleukin 5 (IL-5) is a central cytokine in type 2 inflammation. Alongside multidirectional effects on a wide variety of immune and structural cells involved in inflammation,^22^ IL-5 is a major cytokine responsible for eosinophil maturation, activation, proliferation, migration, and survival in the blood and pulmonary tissue.^22^^,^^23^ Mepolizumab is a humanized monoclonal antibody that specifically binds to IL-5,^24-27^ and inhibition of type 2 inflammation via IL-5 with mepolizumab is a therapeutic avenue for patients with COPD who have elevated BECs.

METREX, METREO, and MATINEE were phase 3 studies that assessed the efficacy and safety of mepolizumab in patients with COPD and a history of exacerbations despite receiving inhaled triple therapy. Patients with a wide spectrum of clinical characteristics and disease severities were studied, including airflow obstruction from Global Initiative for Chronic Obstructive Lung Disease (GOLD) grades 2 to 4, patients with and without chronic bronchitis, and the full spectrum of modified Medical Research Council (mMRC) dyspnea scale scores.^28^^,^^29^ These studies enrolled patients with a wide range of eosinophil levels (from low BEC <150 cells/µL up to ≥300 cells/µL, as defined by the inclusion and exclusion criteria) and with BEC measurements available at multiple timepoints before randomization, including up to 12 months before study start. Across these studies, mepolizumab exhibited consistent efficacy in reducing exacerbation-related outcomes in patients with COPD and an eosinophilic phenotype, defined by screening and/or historical BECs.^28^^,^^29^

This post hoc analysis evaluated exacerbation rates and treatment effects by analyzing pooled data from patients enrolled in the METREX, METREO, and MATINEE studies, according to the degree of BEC variability prior to treatment initiation.

## Methods

### Study design and treatment

This was a pooled post hoc analysis of patients with COPD from 3 phase 3, placebo-controlled, randomized, double-blind, multicenter trials: METREX (ClinicalTrials.gov identifier: NCT02105948), METREO (NCT02105961), and MATINEE (NCT04133909).^28^^,^^29^

**Figure 1:**
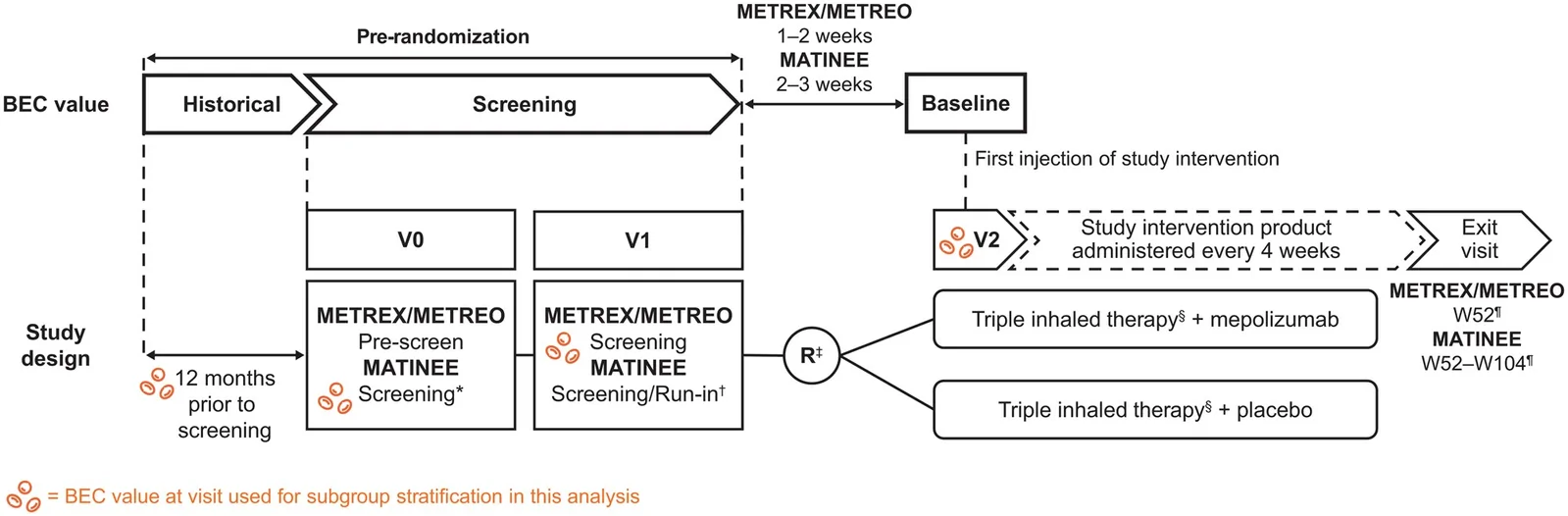
Summary of study design. *In MATINEE, patients were required to have BEC ≥300 cells/μL at screening V0 to proceed to V1, alongside a documented historical BEC ≥150 cells/μL in the 12 months prior to V0. ^†^In MATINEE, patients with no historical BEC ≥150 cells/μL were required to have BEC ≥150 cells/μL at V1. ^‡^Randomization criteria were assessed at W0 (V2), prior to randomization. ^§^Patients had to have received ≥3 months (MATINEE) or ≥12 months (METREX and METREO) SoC background therapy (defined as ICS-based triple therapy consisting of dual long-acting bronchodilators plus ICS [fluticasone propionate ≥500 μg/day or equivalent + LABA + LAMA] unless there was documentation of safety or intolerance issues related to LABA or LAMA) immediately prior to screening; all patients continued SoC background therapy throughout their participation in the trial. ^¶^METREX/METREO were studies of fixed 52-week duration; patients enrolled in METREX/METREO therefore all had data available up to W52. For MATINEE, due to the lower frequency of COPD exacerbations globally during the COVID-19 pandemic, the initial 52-week fixed trial duration was extended to a 104-week variable duration following regulatory approval (Protocol Amendment 6, December 6, 2021); patients enrolled in MATINEE therefore could have data available up to W104. Abbreviations: BEC, blood eosinophil count; COPD, chronic obstructive pulmonary disease; ICS, inhaled corticosteroid; LABA, long-acting β_2_-agonist; LAMA, long-acting muscarinic antagonist; R, randomization; SoC, standard of care; V, visit; W, week.

METREX, METREO, and MATINEE study designs have been previously described in full^28^^,^^29^ and are summarized in Figure 1. In brief, METREX and METREO assessed mepolizumab subcutaneous (SC) injections (100 mg in METREX, 100 or 300 mg in METREO) versus placebo every 4 weeks for 52 weeks.^28^ MATINEE assessed mepolizumab 100 mg versus placebo every 4 weeks over a 104-week variable duration.^29^ In all studies, BEC was assessed at 3 timepoints before first dose of the investigational product: “historical” information in the 12 months prior to screening, “screening” value, and a “baseline” measure on the day of randomization (prior to the first injection of the investigational product). BEC measurements during screening and baseline were taken at stable state (ie, not within 14 days of an exacerbation). Screening and baseline BEC values were analyzed at a central laboratory, while historical BECs were analyzed locally. This analysis focused on patients who had received either ­mepolizumab 100 mg SC or placebo.

METREX, METREO, and MATINEE were conducted in accordance with the ethical principles of the Declaration of Helsinki, the International Conference on Harmonisation Good Clinical Practice Guidelines, and the applicable country-specific regulatory requirements. All patients provided written informed consent.

### Patients

Key patient eligibility criteria have been previously described^28^^,^^29^ and are summarized in the Supplementary Methods and Table S1.

In terms of BEC criteria at enrollment, patients in METREX were enrolled regardless of previous BEC measurements but were stratified at randomization according to a high BEC stratum (defined as BEC ≥150 cells/µL at screening and/or ≥300 cells/µL in the previous year) or a low BEC stratum (BEC <150 cells/µL at screening and no evidence of ≥300 cells/µL in the previous year). In METREO, only patients with BEC ≥150 cells/µL at screening and/or ≥300 cells/µL in the previous year were eligible for inclusion.^28^ Patients in MATINEE were required to have BEC of ≥300 cells/µL at screening and ≥150 cells/µL in the 12 months prior to screening or 14 to 21 days before randomization.^29^

### Endpoints and assessments

**Figure 2:**
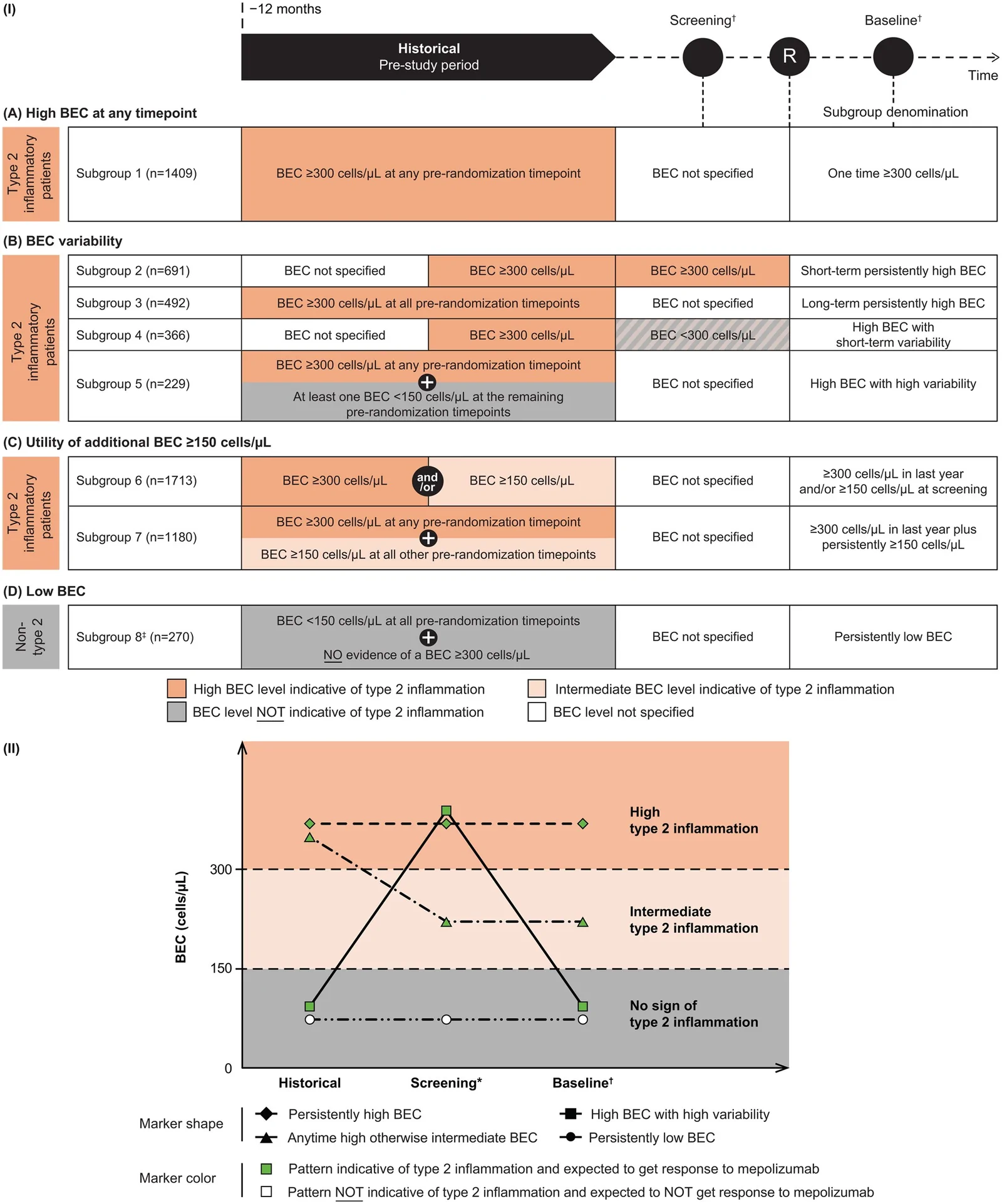
(I) Subgroups used for the analysis were categorized as (A) High BEC at any timepoint, (B) BEC variability, (C) Utility of having an additional BEC measurement ≥150 cells/μL in patients with a record of high BEC (≥300 cells/μL), and (D) Low BEC, resulting in BEC signatures illustrated in panel (II). Subgroups are not mutually exclusive. *Visit 0 or visit 1. ^†^Visit 2. ^‡^Includes only patients from METREX, for whom data are available up until week 52 only. Abbreviations: BEC, blood eosinophil count; R, randomization.

The primary outcome for this pooled analysis was the annualized rate of moderate or severe COPD exacerbations. Secondary outcomes included time to first moderate or severe exacerbation, annualized rate of exacerbations requiring an emergency department (ED) visit and/or hospitalization, time to first exacerbation requiring an ED visit and/or hospitalization, the annualized rate of severe exacerbations, and time to first severe exacerbation. As illustrated in Figure 2-I, outcomes were assessed in non–mutually exclusive subgroups according to high BEC assessed by a single measurement (subgroup 1); BEC variability (subgroups 2 and 3: short- and long-term persistently high BEC, respectively; ­subgroups 4 and 5: high BEC with short-term and high variability, respectively); the utility of having an additional BEC measurement ≥150 cells/µL in patients with a record of high BEC (≥300 cells/µL) (subgroup 6: high BEC in the last year and/or intermediate BEC at screening; subgroup 7: one record of high BEC in the last year and persistently intermediate otherwise); or having persistently low BEC (subgroup 8). Subgroups were defined following expert counsel, using the available BEC measurement timepoints. Resulting BEC signatures are illustrated in Figure 2-II. All outcomes were reported over the 104-week study period, except in subgroup 8 (BEC persistently <150 cells/µL), which only included patients from METREX, who only had data available up to week 52. See the Supplementary Methods for additional information.

### Sample size and statistical analysis

The primary analysis population for this pooled analysis included all patients from the modified intention-to-treat populations from METREX, METREO, and MATINEE who received mepolizumab 100 mg SC or placebo.

Statistical analysis methods are detailed in the Supplementary Methods.

## Results

### Patient population

This pooled analysis included 2089 patients from the METREX (*n* = 836), METREO (*n* = 449), and MATINEE (*n* = 804) trials. Of these, 1043 (49.9%) received mepolizumab and 1046 (50.1%) received placebo. Baseline demographics and clinical characteristics were balanced across treatment arms (Table S2).

Subgroup distributions are described in Figure 2-I. Baseline demographics and clinical characteristics by subgroups are presented in Table 1. While BEC at screening understandably differed between subgroups, characteristics such as history of severe exacerbations, severity of airflow obstruction, presence/absence of symptoms of chronic bronchitis, mMRC score at screening, and St. George’s Respiratory Questionnaire total score at screening were balanced (Table 1).

**Table 1:** Baseline demographics by subgroup category.

	(A) High BEC at any timepoint		**(B) BEC variability**							
	Subgroup 1: One time ≥300 cells/µL (n = 1409)		Subgroup 2: Short-term persistently high BEC (n = 691)		Subgroup 3: Long-term persistently high BEC (n = 492)		**Subgroup 4: High BEC with short-term variability (n = 366)**		Subgroup 5: High BEC with high variability (n = 229)	
	Mepolizumab 100 mg SC (n = 699)	Placebo (n = 710)	Mepolizumab 100 mg SC (n = 342)	Placebo (n = 349)	Mepolizumab 100 mg SC (n = 242)	Placebo (n = 250)	Mepolizumab 100 mg SC (n = 188)	Placebo (n = 178)	Mepolizumab 100 mg SC (n = 116)	Placebo (n = 113)
**Age, y, mean (SD)**	65.8 (8.5)	65.8 (8.3)	65.8 (8.5)	65.2 (8.5)	65.4 (8.4)	65.6 (8.4)	66.8 (8.1)	66.6 (7.2)	64.8 (8.8)	66.4 (7.5)
**Female sex, No. (%)**	241 (34)	232 (33)	108 (32)	114 (33)	68 (28)	82 (33)	71 (38)	63 (35)	47 (41)	35 (31)
**Smoking history, No. (%)**										
**Never**	6 (<1)	8 (1)	1 (<1)	4 (1)	0	2 (1)	0	3 (2)	3 (3)	0
**Currently**	192 (27)	199 (28)	94 (27)	98 (28)	69 (29)	69 (28)	53 (28)	53 (30)	21 (18)	28 (25)
**Formerly**	501 (72)	503 (71)	247 (72)	247 (71)	173 (71)	179 (72)	135 (72)	122 (69)	92 (79)	85 (75)
**Duration of COPD, y, mean (SD)**	9.6 (6.7)	9.4 (6.0)	9.5 (6.5)	9.4 (5.9)	9.6 (6.5)	9.3 (6.1)	10.8 (7.4)	9.5 (5.4)	8.5 (5.5)	10.1 (6.4)
**BEC at screening, cells/µL, geometric mean (logSD)**	370 (0.716)	380 (0.725)	510 (0.393)	510 (0.420)	530 (0.406)	530 (0.439)	410 (0.288)	410 (0.307)	170 (1.179)	170 (1.161)
**Severe exacerbations in the 12 mo prior to screening, No. (%)**										
**0**	504 (72)	521 (73)	247 (72)	261 (75)	179 (74)	195 (78)	141 (75)	135 (76)	84 (72)	78 (69)
**≥1**	195 (28)	189 (27)	95 (28)	88 (25)	63 (26)	55 (22)	47 (25)	43 (24)	32 (28)	35 (31)
**Symptoms of chronic bronchitis^a^**										
**Patients with assessment, No.**	680	699	334	341	236	244	180	177	112	111
**Presence, No. (%)**	449 (66)	463 (66)	234 (70)	224 (66)	173 (73)	160 (66)	115 (64)	122 (69)	68 (61)	76 (68)
**Absence, No. (%)**	231 (34)	236 (34)	100 (30)	117 (34)	63 (27)	84 (34)	65 (36)	55 (31)	44 (39)	35 (32)
**Severity of airflow limitation^b^, No. (%)**										
**Moderate: ≥50% to <80% predicted**	286 (51)	287 (40)	150 (44)	147 (42)	103 (43)	107 (43)	75 (40)	74 (42)	45 (39)	36 (32)
**Severe: ≥30% to <50% predicted**	309 (44)	313 (44)	153 (45)	153 (44)	112 (46)	108 (43)	84 (45)	74 (42)	51 (44)	55 (49)
**Very severe: <30% predicted**	98 (14)	103 (15)	37 (11)	45 (13)	24 (10)	33 (13)	28 (15)	28 (16)	19 (16)	21 (19)
**mMRC score at screening^c^, No. (%)**										
**Patients with assessment, No.**	695	708	339	348	239	249	187	177	115	113
**<2**	135 (19)	154 (22)	77 (23)	82 (24)	54 (23)	61 (24)	30 (16)	36 (20)	25 (22)	19 (17)
**≥2**	560 (81)	554 (78)	262 (77)	266 (76)	185 (77)	188 (76)	157 (84)	141 (80)	90 (78)	94 (83)
**SGRQ total score at baseline^d^**										
**Patients with assessment, No.**	678	698	332	340	235	243	180	177	112	111
**Mean (SD)**	54.5 (17.4)	53.9 (17.3)	54.6 (17.1)	53.4 (17.6)	56.0 (17.4)	52.2 (17.6)	54.4 (17.9)	55.3 (17.2)	54.3 (16.4)	55.9 (18.4)

	**(C) Utility of additional BEC ≥150 cells/µL**				**(D) Low BEC**	
	Subgroup 6: ≥300 cells/µL in last year and/or ≥150 cells/µL at screening (n = 1713)		Subgroup 7: ≥300 cells/µL in last year plus persistently ≥150 cells/µL (n = 1180)		Subgroup 8: Persistently low BEC (n = 270)	
	Mepolizumab 100 mg SC (n = 857)	Placebo (n = 856)	Mepolizumab 100 mg SC (n = 583)	Placebo (n = 597)	Mepolizumab 100 mg SC (n = 138)	Placebo (n = 132)
**Age, y, mean (SD)**	65.7 (8.5)	65.8 (8.3)	66.0 (8.5)	65.7 (8.4)	66.3 (9.0)	64.8 (9.1)
**Female sex, No. (%)**	302 (35)	275 (32)	194 (33)	197 (33)	53 (38)	59 (45)
**Smoking history, No. (%)**						
**Never**	12 (1)	13 (2)	3 (<1)	8 (1)	5 (4)	11 (8)
**Currently**	228 (27)	246 (29)	171 (29)	171 (29)	31 (22)	34 (26)
**Formerly**	617 (72)	597 (70)	409 (70)	418 (70)	102 (74)	87 (66)
**Duration of COPD, y, mean (SD)**	9.6 (6.7)	9.4 (6.0)	9.8 (6.9)	9.3 (5.9)	8.5 (5.1)	9.4 (7.2)
**BEC at screening, cells/µL, geometric mean (logSD)**	330 (0.687)	340 (0.679)	430 (0.432)	440 (0.472)	60 (0.680)	60 (0.746)
**Severe exacerbations in the 12 mo prior to screening, No. (%)**						
**0**	617 (72)	618 (72)	420 (72)	443 (74)	101 (73)	95 (72)
**≥1**	240 (28)	238 (28)	163 (28)	154 (26)	37 (27)	37 (28)
**Symptoms of chronic bronchitis^a^**						
**Patients with assessment, No.**	836	841	568	588	137	127
**Presence, No. (%)**	530 (63)	548 (65)	381 (67)	387 (66)	73 (53)	69 (54)
**Absence, No. (%)**	306 (37)	293 (35)	187 (33)	201 (34)	64 (47)	58 (46)
**Severity of airflow limitation^b^, No. (%)**						
**Moderate: ≥50% to <80% predicted**	337 (39)	337 (39)	241 (41)	251 (42)	51 (37)	43 (33)
**Severe: ≥30% to <50% predicted**	388 (45)	377 (44)	258 (44)	258 (43)	59 (43)	64 (48)
**Very severe: <30% predicted**	124 (14)	135 (16)	79 (14)	82 (14)	27 (20)	24 (18)
**mMRC score at screening^c^, No. (%)**						
**Patients with assessment, No.**	852	854	580	595	138	132
**<2**	174 (20)	179 (21)	110 (19)	135 (23)	25 (18)	21 (16)
**≥2**	678 (80)	675 (79)	470 (81)	460 (77)	113 (82)	111 (84)
**SGRQ total score at baseline^d^**						
**Patients with assessment, No.**	834	840	566	587	137	127
**Mean (SD)**	54.1 (17.6)	54.3 (16.9)	54.6 (17.6)	53.5 (17.1)	54.0 (17.4)	54.9 (16.1)

Subgroups are not mutually exclusive.

Abbreviations: BEC, blood eosinophil count; COPD, chronic obstructive pulmonary disease; FEV_1_, forced expiratory volume in 1 second; GOLD, Global Initiative for Chronic Obstructive Lung Disease; mMRC, modified Medical Research Council; NHANES, National Health and Nutrition Examination Survey; SC, subcutaneous; SD, standard deviation; SGRQ, St. George’s Respiratory Questionnaire.

a Symptoms of chronic bronchitis were assessed on the basis of responses at baseline to items on the SGRQ related to cough and sputum.

b The severity of airflow obstruction was assessed based on the postbronchodilator FEV_1_ at screening according to the GOLD guidelines for COPD. A postbronchodilator FEV_1_ of ≥80% of the predicted normal value was considered to indicate mild airflow obstruction; ≥50% to <80%, moderate; ≥30% to <50%, severe; and <30%, very severe. FEV_1_ predicted values were calculated using NHANES III reference equations. In total, 17 patients (9 mepolizumab, 8 placebo) had mild airflow obstruction (GOLD grade 1 [mild]: FEV_1_ ≥80% predicted) as FEV_1_ = 80% predicted was not excluded from the study.

c Grades on the mMRC dyspnea scale range from 0 to 4, with higher grades indicating more severe dyspnea.

d Total scores on the SGRQ range from 0 to 100, with higher scores indicating worse health status.

### Single high BEC measurement at any timepoint

**Figure 3:**
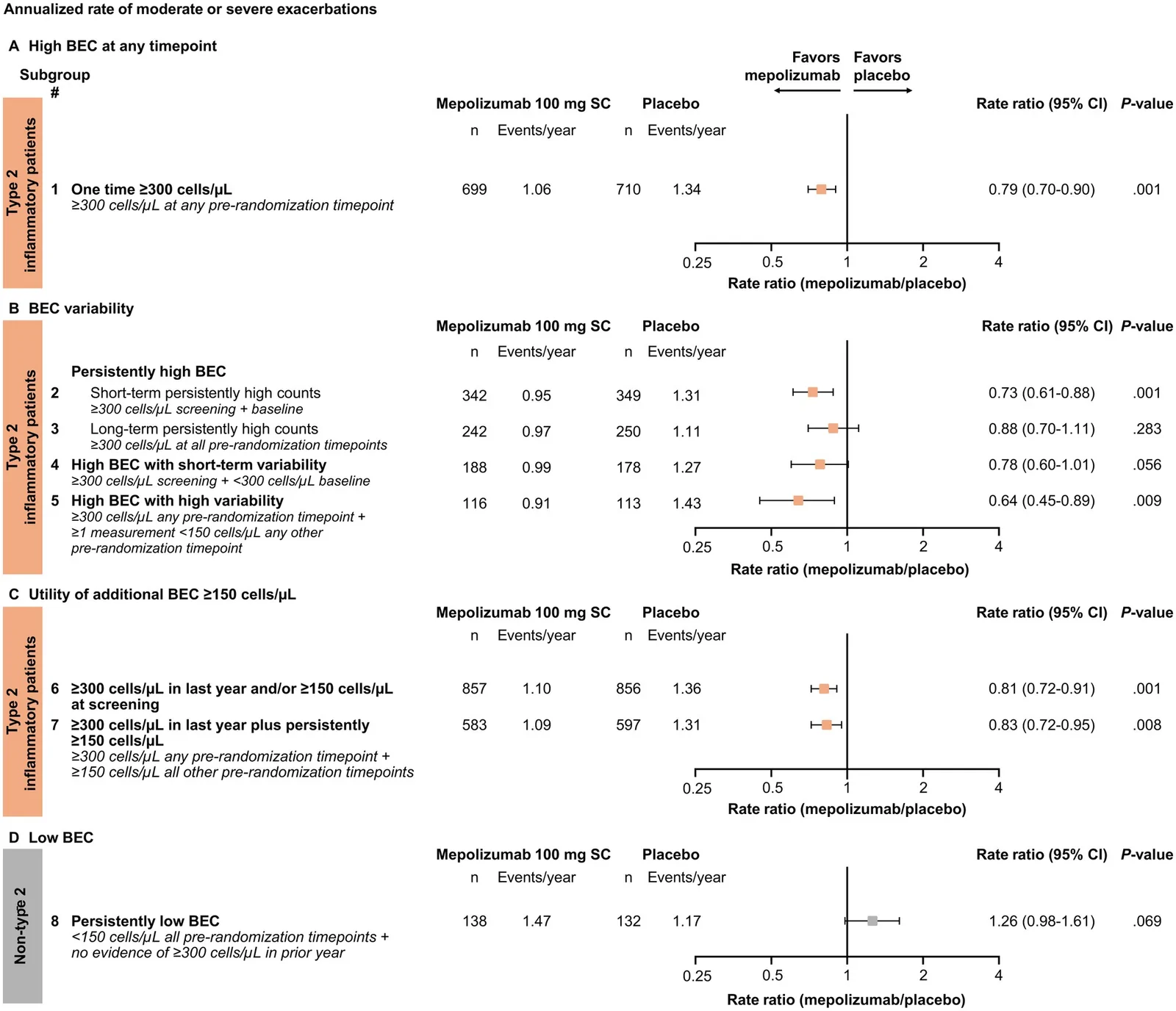
Primary endpoint: annualized rate of moderate or severe exacerbations by BEC variability. The annualized rates of moderate or severe exacerbations were reduced with mepolizumab in all patients with type 2 inflammation, regardless of (A) having a single BEC measurement ≥300 cells/μL, (B) BEC variability and (C) presence of an additional BEC measurement ≥150 cells/µL, compared with placebo. Patients with sporadic BEC measurements ≥300 cells/µL showed the greatest treatment effect, and those with BEC persistently ≥300 cells/µL the lowest. There were no treatment benefits in (D) patients without type 2 inflammation. Subgroups are not mutually exclusive. The analysis was performed using a negative binomial regression model with covariates of treatment group, study, smoking status, baseline % predicted FEV_1_, number of exacerbations in the prior year (as an ordinal variable), and geographic region (as defined for the study/integrated analysis) with log of time in the on- and off-treatment periods as an offset variable. Data were assessed for up to week 104, except for subgroup 8, which included only patients from METREX, for whom data were only available up to week 52. Abbreviations: BEC, blood eosinophil count; CI, confidence interval; FEV_1_, forced expiratory volume in 1 second; SC, subcutaneous.

**Figure 4:**
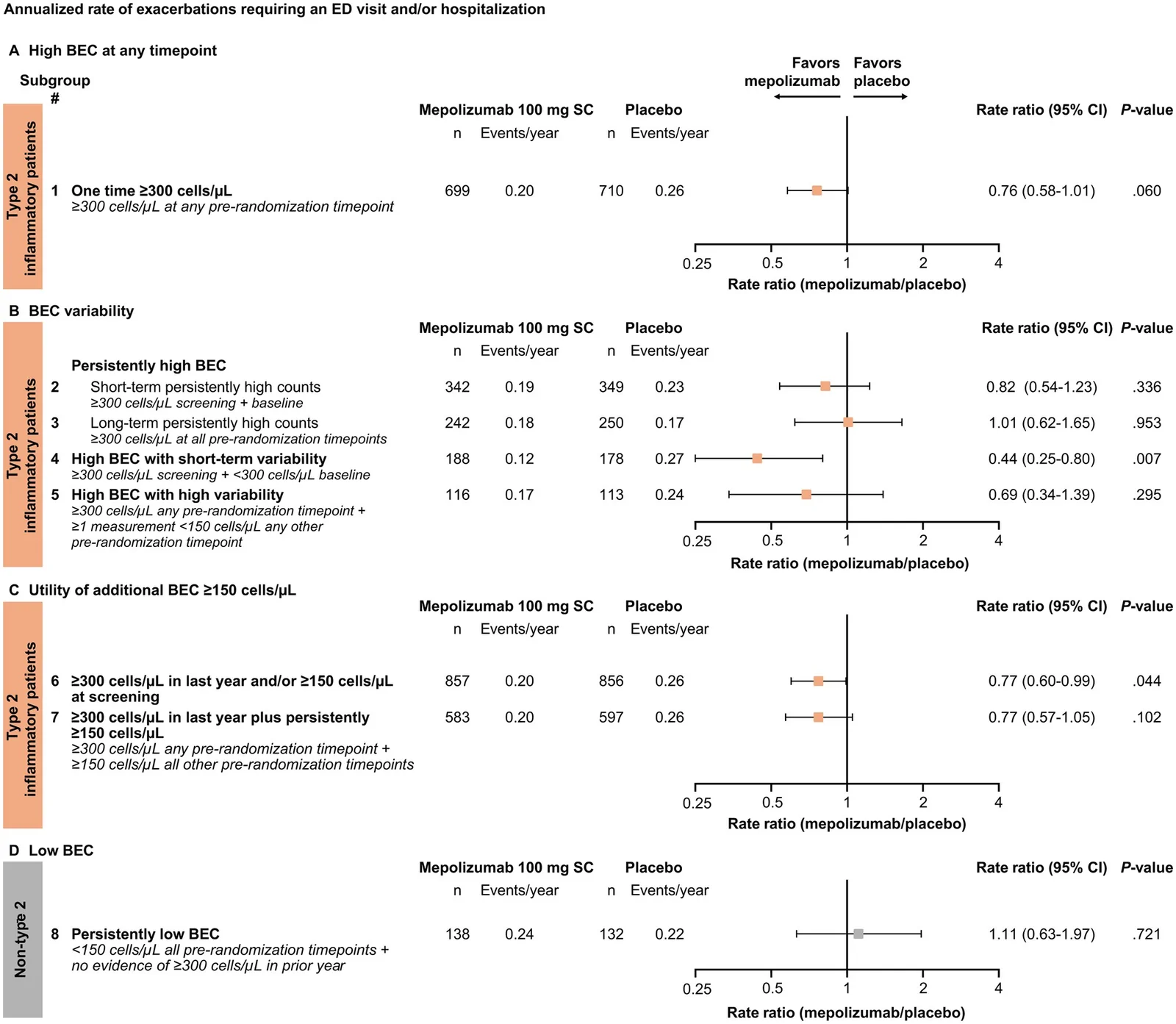
Secondary endpoint: annualized rate of exacerbations requiring ED visit and/or hospitalization by BEC variability. The annualized rates of exacerbations requiring an ED visit and/or hospitalization trended toward a reduction with mepolizumab versus placebo in patients with type 2 inflammation regardless of (A) having a single BEC measurement ≥300 cells/μL, (B) BEC variability and (C) presence of an additional BEC measurement ≥150 cells/µL, except in those with BEC ≥300 cells/µL at all pre-randomization timepoints. There were no treatment benefits in (D) patients without type 2 inflammation. Subgroups are not mutually exclusive. The analysis was performed using a negative binomial regression model with covariates of treatment group, study, smoking status, baseline % predicted FEV_1_, number of exacerbations in the prior year (as an ordinal variable), and geographic region (as defined for the study/integrated analysis) with log of time in the on- and off-treatment periods as an offset variable. Data were assessed for up to week 104, except in subgroup 8, which included only patients from METREX, for whom data were only available up to week 52. Abbreviations: BEC, blood eosinophil count; CI, confidence interval; ED, emergency department; FEV_1_, forced expiratory volume in 1 second; SC, subcutaneous.

There was a significant 21% reduction in the rate of moderate or severe exacerbations in patients with high BEC measurement on one occasion (subgroup 1; BEC ≥300 cells/µL at any pre-randomization timepoint, *n* = 1409; rate ratio [RR], 0.79 [95% CI, 0.70-0.90]; *P* = .001) (Figure 3). Exacerbations requiring ED visit and/or hospitalization and severe exacerbations trended toward a reduction without reaching statistical significance (Figure 4 and Figure S1).

The hazard of first moderate or severe exacerbation was significantly reduced by 28% (*P* <.001) in subgroup 1 (Table 2 and Figure S2). The hazards of first exacerbation requiring ED visit and/or hospitalization (Table 2 and Figure S3), as well as first severe exacerbation (Table 2), trended numerically toward a reduction without reaching statistical significance.

**Table 2:** Secondary endpoint: time to first exacerbation.

Subgroup	Exacerbation	**Mepolizumab 100 mg SC (*n*** = **1043)**	**Placebo (*n*** = **1046)**
**(A) High BEC at any timepoint**			
** Subgroup 1: One-time ≥300 cells/µL**	**Moderate or severe exacerbations**		
	Probability of an exacerbation by week 104^a^, %	68.4	74.1
	HR mepolizumab/placebo (95% CI)	0.72 (0.63-0.83)	
	*P* value	<.001	
	**Exacerbations requiring an ED visit and/or hospitalization**		
	Probability of an exacerbation by week 104^a^, %	20.7	26.4
	HR mepolizumab/placebo (95% CI)	0.78 (0.61-1.00)	
	*P* value	.055	
	**Severe exacerbations**		
	Probability of an exacerbation by week 104^a^, %	18.4	23.2
	HR mepolizumab/placebo (95% CI)	0.78 (0.60-1.02)	
	*P* value	.071	
**(B) BEC variability**			
** Subgroup 2: Short-term persistently high BEC**	**Moderate or severe exacerbations**		
	Probability of an exacerbation by week 104^a^, %	62.6	75.7
	HR mepolizumab/placebo (95% CI)	0.64 (0.52-0.78)	
	*P* value	<.001	
	**Exacerbations requiring an ED visit and/or hospitalization**		
	Probability of an exacerbation by week 104^a^, %	21.3	27.2
	HR mepolizumab/placebo (95% CI)	0.80 (0.56-1.14)	
	*P* value	.223	
	**Severe exacerbations**		
	Probability of an exacerbation by week 104^a^, %	16.8	23.6
	HR mepolizumab/placebo (95% CI)	0.76 (0.51-1.14)	
	*P* value	.187	
** Subgroup 3: Long-term persistently high BEC**	**Moderate or severe exacerbations**		
	Probability of an exacerbation by week 104^a^, %	63.0	69.2
	HR mepolizumab/placebo (95% CI)	0.78 (0.61-0.99)	
	*P* value	.041	
	**Exacerbations requiring an ED visit and/or hospitalization**		
	Probability of an exacerbation by week 104^a^, %	20.8	24.7
	HR mepolizumab/placebo (95% CI)	0.88 (0.57-1.35)	
	*P* value	.550	
	**Severe exacerbations**		
	Probability of an exacerbation by week 104^a^, %	16.0	21.5
	HR mepolizumab/placebo (95% CI)	0.79 (0.49-1.30)	
	*P* value	.360	
** Subgroup 4: High BEC with short-term variability**	**Moderate or severe exacerbations**		
	Probability of an exacerbation by week 104^a^, %	78.5	74.2
	HR mepolizumab/placebo (95% CI)	0.79 (0.60-1.04)	
	*P* value	.097	
	**Exacerbations requiring an ED visit and/or hospitalization**		
	Probability of an exacerbation by week 104^a^, %	15.9	25.3
	HR mepolizumab/placebo (95% CI)	0.53 (0.31-0.90)	
	*P* value	.020	
	**Severe exacerbations**		
	Probability of an exacerbation by week 104^a^, %	17.6	23.7
	HR mepolizumab/placebo (95% CI)	0.61 (0.35-1.05)	
	*P* value	.076	
** Subgroup 5: High BEC with high variability**	**Moderate or severe exacerbations**		
	Probability of an exacerbation by week 104^a^, %	79.7	72.7
	HR mepolizumab/placebo (95% CI)	0.55 (0.39-0.79)	
	*P* value	.002	
	**Exacerbations requiring an ED visit and/or hospitalization**		
	Probability of an exacerbation by week 104^a^, %	21.2	19.4
	HR mepolizumab/placebo (95% CI)	0.92 (0.48-1.74)	
	*P* value	.795	
	**Severe exacerbations**		
	Probability of an exacerbation by week 104^a^, %	18.7	16.6
	HR mepolizumab/placebo (95% CI)	0.82 (0.41-1.64)	
	*P* value	.573	
**(C) Utility of additional BEC ≥150 cells/µL**			
** Subgroup 6: ≥300 cells/µL in last year and/or ≥150 cells/µL at screening**	**Moderate or severe exacerbations**		
	Probability of an exacerbation by week 104^a^, %	70.0	74.6
	HR mepolizumab/placebo (95% CI)	0.78 (0.69-0.88)	
	*P* value	<.001	
	**Exacerbations requiring an ED visit and/or hospitalization**		
	Probability of an exacerbation by week 104^a^, %	20.7	26.7
	HR mepolizumab/placebo (95% CI)	0.78 (0.62-0.97)	
	*P* value	.029	
	**Severe exacerbations**		
	Probability of an exacerbation by week 104^a^, %	18.6	23.7
	HR mepolizumab/placebo (95% CI)	0.78 (0.61-0.99)	
	*P* value	.045	
** Subgroup 7: ≥300 cells/µL in last year plus persistently ≥150 cells/µL**	**Moderate or severe exacerbations**		
	Probability of an exacerbation by week 104^a^, %	68.4	74.1
	HR mepolizumab/placebo (95% CI)	0.76 (0.65-0.88)	
	*P* value	<.001	
	**Exacerbations requiring an ED visit and/or hospitalization**		
	Probability of an exacerbation by week 104^a^, %	20.8	26.9
	HR mepolizumab/placebo (95% CI)	0.78 (0.60-1.02)	
	*P* value	.068	
	**Severe exacerbations**		
	Probability of an exacerbation by week 104^a^, %	18.4	23.5
	HR mepolizumab/placebo (95% CI)	0.78 (0.58-1.05)	
	*P* value	.104	
**(D) Persistently low BEC**			
** Subgroup 8: <150 cells/µL at all pre-randomization timepoints and no evidence of ≥300 cells/µL at any timepoint**	**Moderate or severe exacerbations**		
	Probability of an exacerbation by week 52^a^ ^,^ ^b^, %	64.0	62.5
	HR mepolizumab/placebo (95% CI)	1.19 (0.87-1.62)	
	*P* value	.282	
	**Exacerbations requiring an ED visit and/or hospitalization**		
	Probability of an exacerbation by week 52^a^ ^,^ ^b^, %	21.4	19.0
	HR mepolizumab/placebo (95% CI)	1.20 (0.69-2.09)	
	*P* value	.527	
	**Severe exacerbations**		
	Probability of an exacerbation by week 52^a^, %	16.0	15.2
	HR mepolizumab/placebo (95% CI)	1.10 (0.58-2.07)	
	*P* value	.770	

Subgroups are not mutually exclusive. The analysis was performed using a Cox proportional hazards model with covariates of treatment group, study, smoking status, baseline % predicted FEV_1_, number of exacerbations in the prior year (as an ordinal variable), and geographic region (as defined for the study/integrated analysis).

Abbreviations: BEC, blood eosinophil count; CI, confidence interval; ED, emergency department; FEV_1_, forced expiratory volume in 1 second; HR, hazard ratio; SC, subcutaneous.

a Kaplan–Meier estimate.

b Includes only patients from METREX, for whom data are available up until week 52 only.

### Effect of BEC variability

Patients with short-term persistently high BEC (subgroup 2; ≥300 cells/µL at screening and baseline, *n* = 691) showed a statistically significant 27% reduction in the annualized rate of moderate or severe exacerbations (RR, 0.73 [95% CI, 0.61-0.88]; *P* = .001), whereas patients with long-term persistently high BEC (subgroup 3; ≥300 cells/µL at all pre-randomization timepoints, *n* = 492) showed a nonsignificant 12% reduction (RR, 0.88 [95% CI, 0.70-1.11]; *P* = .283). Patients in subgroup 3 also had the lowest annualized rate of moderate or severe exacerbations across placebo groups, with 1.11 events/year. In contrast, patients with high BEC and short-term variability (subgroup 4; BEC ≥300 cells/µL at screening but <300 cells/µL at baseline, *n* = 366) trended toward a 22% rate reduction in moderate or severe exacerbations (RR, 0.78 [95% CI, 0.60-1.01]; *P* = .056) with mepolizumab. Patients with high BEC and high variability (subgroup 5; any BEC ≥300 cells/µL and ≥ 1 measurement <150 cells/µL pre-randomization, *n* = 229) experienced a significant 36% reduction (RR, 0.64 [95% CI, 0.45-0.89]; *P* = .009), compared with placebo. Patients receiving placebo in subgroup 5 also had the highest rate of moderate or severe exacerbations across all subgroups, with 1.43 events/year (Figure 3).

In subgroup 4, exacerbations requiring ED visit and/or hospitalization (Figure 4) were significantly reduced by 56% (RR, 0.44 [95% CI, 0.25-0.80]; *P* = .007), and severe exacerbations (Figure S1) by 47% (RR, 0.53 [95% CI, 0.29-0.97]; *P* = .038). These outcomes trended toward reductions with mepolizumab versus placebo in subgroups 2 and 5, albeit without reaching statistical significance; there were no differences between treatment groups in subgroup 2 (Figure 4 and Figure S1).

The hazard of first moderate or severe exacerbation up to week 104 was significantly reduced with mepolizumab by 36% in subgroup 2 (*P* <.001), 22% in subgroup 3 (*P* = .041), and 45% (*P* = .002) in subgroup 5 (Table 2 and Figure S2), compared with placebo; subgroup 4 trended toward a reduction but without reaching statistical significance. The hazard of first exacerbation requiring ED visit and/or hospitalization was significantly reduced by 47% (*P* = .020) in subgroup 4; other subgroups trended toward a reduction, albeit without reaching statistical significance (Table 2 and Figure S3).

### Utility of additional BEC measurement ≥150 cells/µL

Patients in subgroup 6, with BEC ≥300 cells/µL in the last year and/or BEC ≥150 cells/µL at screening (*n* = 1713), had a statistically significant 19% reduction in the annualized rate of moderate or severe exacerbations (RR, 0.81 [95% CI, 0.72-0.91]; *P* = .001). In subgroup 7, which included patients who had a BEC ≥300 cells/µL at any pre-randomization timepoint plus all other pre-randomization measurements ≥150 cells/µL (*n* = 1180), there was a significant 17% rate reduction (RR, 0.83 [95% CI, 0.72-0.95]; *P* = .008) (Figure 3).

Exacerbations requiring ED visit and/or hospitalization were significantly reduced by 23% (RR, 0.77 [95% CI, 0.60-0.99]; *P* = .044) in subgroup 6 and trended toward a reduction without reaching statistical significance in subgroup 7 (Figure 4). Severe exacerbations trended similarly across the 2 subgroups, albeit without reaching statistical significance (Figure S1).

Patients receiving mepolizumab in subgroup 6 experienced statistically significant reductions of 22% in the hazards of first moderate or severe exacerbation (*P* <.001) (Table 2 and Figure S2), exacerbation requiring an ED visit and/or hospitalization (*P* = .029) (Table 2 and Figure S3), and severe exacerbation (*P* = .045) up to week 104, versus placebo (Table 2). The hazard of first moderate or severe exacerbation was also significantly reduced by 24% (*P* <.001) in subgroup 7 (Table 2 and Figure S2). The hazard of first exacerbation requiring ED visit and/or hospitalization (Table 2 and Figure S2), as well as of first severe exacerbation, trended toward a reduction in subgroup 7, but without reaching statistical significance (Table 2).

### Patients with persistently low BEC

Patients with persistently low BEC (subgroup 8; <150 cells/µL at all pre-randomization timepoints and no evidence of ≥300 cells/µL in the prior year, *n* = 270) showed no improvements with mepolizumab versus placebo in any of the assessed exacerbation outcomes (Figures 3 and 4, Table 2, and Figures S1-S3).

## Discussion

This pooled post hoc analysis of the phase 3 METREX, METREO, and MATINEE trials is the first to demonstrate the variability of type 2 inflammation over time (characterized by BEC up to 12 months before randomization; Figure 2-II) and its impact on the efficacy of a therapeutic intervention in COPD. The subgroups including patients with BECs ≥300 cells/µL at any point prior to randomization, even if BECs were not consistently high throughout the pre-randomization period, experienced a significant reduction in the rates of moderate or severe exacerbations (subgroups 1 and 7). In contrast, patients with BEC <150 cells/µL at all pre-randomization timepoints and no evidence of BEC ≥300 cells/µL in the prior year experienced no benefits in any of the studied exacerbation outcomes with mepolizumab (subgroup 8). These findings indicate that a single BEC measurement ≥300 cells/µL (including historical) is sufficient to identify COPD subgroups who experience a significant rate reduction in moderate or severe exacerbations with mepolizumab.

Additionally, patients with BEC ≥150 cells/µL at screening and/or a BEC measurement ≥300 cells/µL in the 12 months prior to randomization (subgroup 6) exhibited significant rate reductions and delays in experiencing the first moderate or severe COPD exacerbation, and exacerbations requiring ED visit and/or hospitalization. The rate reductions in moderate or severe exacerbations were similar to subgroup 1 (one time ≥300 cells/µL), indicating that the additional BEC measurement performed in subgroup 6 did not further enrich the population with individuals with potential for a greater treatment response. This further supports the concept that a single BEC measurement ≥300 cells/µL, regardless of timepoint, can identify potential responders to treatment with mepolizumab. Interestingly, in patients with at least one BEC measurement ≥300 cells/µL and at least one measurement <150 cells/µL (high BEC with high variability; subgroup 5), the reduction in moderate or severe exacerbations was most pronounced with a significant reduction of 36%. Notably, this subgroup also exhibited the highest rate of moderate or severe exacerbations across subgroups in its placebo group (1.43 events/year). In contrast, the subgroup with BECs ≥300 cells/µL at all timepoints prior to randomization (long-term persistently high BEC; subgroup 3) had the lowest reduction in moderate or severe exacerbations among the analyzed type 2 inflammatory patients (12%, not statistically significant); this subgroup also had the lowest moderate or severe exacerbation rate across all placebo groups (1.11 events/year). Considering that elevated BECs are associated with increased risks of COPD exacerbations,^3^^,^^4^ it may be that patients with high BEC and high variability (subgroup 5) correspond to patients who experience sudden large rises in their BEC, increasing their exacerbation probability, which is supported by the exacerbation rates observed in the placebo group. The association between eosinophil variability and COPD outcomes was also observed in a recent prospective longitudinal cohort from the Chinese Clinical Trial Registry, in which patients with COPD and unstable BEC experienced significantly higher annualized rates of moderate-to-severe exacerbations than those with persistently low BEC, whereas those with persistently high BEC did not.^30^ This is also coherent with the results of a large real-world cohort of patients with asthma, where BEC variability over time was a better predictor for hospital episodes than individual BEC measurements,^31^ though past results from other studies in COPD may indicate a different pattern.^3^ Mepolizumab may help prevent such sudden increases in BEC from occurring and therefore reduce the risk of moderate or severe COPD exacerbations in such patients. Together, these results suggest that BEC fluctuations in patients who sporadically experience BECs ≥300 cells/µL may not negatively impact mepolizumab efficacy and may even lead to a superior response compared with patients who persistently have BEC ≥300 cells/µL.

In this analysis, we also observed consistent reductions in exacerbation rates between patients showing high BEC with short-term variability (subgroup 4) or short-term persistently high BEC (subgroup 2). Patients with BEC ≥300 cells/µL at screening responded similarly regardless of whether they had a BEC measurement ≥300 cells/µL or <300 cells/µL at baseline, with 27% and 22% reductions in the rates of moderate or severe exacerbations versus placebo. These data suggest that short-term BEC variations around the 300 cells/µL threshold do not influence the efficacy of mepolizumab.

This analysis showed some patients may have a BEC measurement <300 cells/µL (or even <150 cells/µL) at certain timepoints, but with previous values ≥300 cells/µL and still show significant benefits in exacerbation-related outcomes from treatment with mepolizumab. This suggests that repeated testing of BEC in patients with COPD is the appropriate method to identify patients with type 2 inflammation in clinical practice, confirming observations previously reported in the literature.^14^ Single readings may not always identify an individual with type 2 inflammation (ie, a false-negative result); these patients may be classified as not having type 2 inflammation and denied referral to biologics such as mepolizumab, despite potentially benefiting from them.

Among patients with persistently low eosinophil levels (subgroup 8; <150 cells/µL at all pre-randomization timepoints and no evidence of ≥300 cells/µL at any timepoint), there were no significant improvements in any of the exacerbation outcomes studied. These findings support the approved US indication for ­mepolizumab in COPD, which is patients with inadequately controlled COPD and an eosinophilic phenotype, defined as BEC ≥150 cells/µL or a historical record of BEC ≥300 cells/µL.^25^

Alongside the previously described limitations of METREX, METREO, and MATINEE,^28^^,^^29^ there were also limitations to this secondary analysis, namely linked to BEC. The 3 studies were designed and powered to detect differences in populations with predefined BEC thresholds (ie, high/low BEC stratum in METREX, eosinophilic phenotype in METREO, and BEC ≥300 cells/µL in MATINEE); the present analysis was therefore exploratory, and the studies were not originally powered to detect differences in subgroups split by BEC variability. Consequently, no adjustments for multiplicity were applied in this analysis; the results presented, including 95% CIs and *P* values, should therefore be interpreted with this limitation in mind. Additionally, only one study (METREX) included patients with BEC <150 cells/µL, comprising the “persistently low BEC” subgroup (subgroup 8), which may affect the generalizability of the results to this nonnegligible subset of patients with COPD. Screening and baseline BEC values were centrally read, while historical BECs were collected locally, which may result in differences in interpretation. Historical BECs were only recorded in the 12 months preceding screening, so it is unknown whether BECs from longer timeframes (such as 2-3 years prior) would have impact on the study’s results. Another consideration is that the studies required that BECs be obtained outside of exacerbations, whereas, in real-world clinical practice, many BEC samples may be taken during acute exacerbations. Nonetheless, this analysis provided high-quality multicenter pooled data across many patients with multiple BEC measurements, including long-term historical measurements. The patient population was well-characterized and excluded patients with other main causes of eosinophilia, while still covering a wide spectrum of patients with COPD by including multiple GOLD stages ranging from moderate to very severe airflow obstruction levels, regardless of presence or absence of chronic bronchitis, emphysema, or both, and covering the full range of mMRC dyspnea scale grades. While the study involved a high number of subgroups, increasing the chances of false discovery, a consistent treatment effect was observed across subgroup groupings (Figure 2A-D), supporting the robustness of the results. This study’s results therefore provide reliable guidance on the efficacy of mepolizumab in patients with variable BECs.

In conclusion, this analysis shows consistent reduction of exacerbations among patients with COPD and type 2 inflammation, indicated by a single reading of high BEC in the last year preceding therapy initiation. These data support the use of mepolizumab in patients who have shown signs of type 2 inflammation as characterized by elevated BEC but are not necessarily persistently eosinophilic. These results support routine BEC testing in clinical practice to properly identify individuals with type 2 inflammation. In turn, this would inform decision making regarding initiating biologic therapy in patients with COPD at risk of exacerbation despite inhaled triple therapy. Further investigation is warranted to identify whether the effects of BEC variability observed on exacerbation-related outcomes in this analysis also apply to other outcomes such as lung function or quality of life.

## Supplementary Material

aamag288_Supplementary_Data

## Data Availability

Anonymized individual patient data and study documents can be requested for further research at https://www.gsk-studyregister.com/en/. All authors had full access to the data upon request and had final responsibility for the decision to submit for publication.
