# Supplementary material for: Mepolizumab efficacy in chronic obstructive pulmonary disease: insights from longitudinal patterns of blood eosinophil counts and their variability across 3 clinical trials
Source: Am J Respir Crit Care Med. 2026 Jun 9;212(9):2022–35. doi: 10.1093/ajrccm/aamag288 (PMC13519312; doi:10.1093/ajrccm/aamag288)
Supplement: Supplementary file 1 [file aamag288_supplementary_data.zip › Martinez_COI form.docx]

| ICMJE DISCLOSURE FORM | |
| --- | --- |
| **Date:** | 12/1/2025 |
| **Your Name:** | Fernando J. Martinez |
| **Manuscript Title:** | Mepolizumab in COPD with Type 2 Inflammation: Efficacy Across Variable Eosinophil Patterns |
| **Manuscript Number (if known):** | Click or tap here to enter text. |
| In the interest of transparency, we ask you to disclose all relationships/activities/interests listed below that are related to the content of your manuscript. “Related” means any relation with for-profit or not-for-profit third parties whose interests may be affected by the content of the manuscript. Disclosure represents a commitment to transparency and does not necessarily indicate a bias. If you are in doubt about whether to list a relationship/activity/interest, it is preferable that you do so.  The author’s relationships/activities/interests should be defined broadly. For example, if your manuscript pertains to the epidemiology of hypertension, you should declare all relationships with manufacturers of antihypertensive medication, even if that medication is not mentioned in the manuscript.  In item #1 below, report all support for the work reported in this manuscript without time limit. For all other items, the time frame for disclosure is the past 36 months. | |

|  | | | **Name all entities with whom you have this relationship or indicate none (add rows as needed)** | **Specifications/Comments (e.g., if payments were made to you or to your institution)** |
| --- | --- | --- | --- | --- |
| **Time frame: Since the initial planning of the work** | | | | |
| **1** | All support for the present manuscript (e.g., funding, provision of study materials, medical writing, article processing charges, etc.)  **No time limit for this item.** | | ☒ GSK \| Supported the parent studies.; GSK \| Manuscript development funded by GSK with medical writing support from Avalere Health; \| Click the tab key to add additional rows. | |
| **Time frame: past 36 months** | | | | |
| **2** | | Grants or contracts from any entity (if not indicated in item #1 above). | ☐ \| **None** AstraZeneca \| Received grant support, AZ is a partner in the SPIROMICS program and partnered in the NHLBI CAPTURE validation study.; Chiesi \| Received in kind grant support. Chiesi is a partner in the SPIROMICS program.; GSK \| Received grant support, GSK is a partner in the SPIROMICS program and partnered in the NHLBI CAPTURE validation study.; Sanofi/Regeneron \| Received in kind grant support, Sanofi/Regeneron are partners of the SPIROMICS program; NHLBI - AstraZeneca*,* Chiesi, Boehringer-Ingelheim, GlaxoSmithKline, Novartis, and Sanofi/Regeneron \| Payments made to the COPD Foundation for partnership in SPIROMICS and/or CAPTURE | |
| **3** | | Royalties or licenses | ☐ \| **None** | |
| **4** | | Consulting fees | ☐ \| **None** AstraZeneca \| Received travel support, AstraZeneca is a partner of the SPIROMICS program and partnered in the NHLBI CAPTURE validation study; Chiesi \| COPD Steering Committee and Global Advisory Board. Received travel support; GSK \| Received travel support, GSK is a partner of the SPIROMICS program and partnered in the NHLBI CAPTURE validation study; DevPro \| COPD Steering committee. No fees.; Roche \| COPD consultation; no fees; Sanofi/Regeneron \| COPD Steering Committee and Advisory Board. Received consulting fees, Sanofi/Regeneron are partners of the SPIROMICS program; UpToDate \| Received payment or honoraria for COPD CME | |
| **5** | | Payment or honoraria for lectures, presentations, speakers bureaus, manuscript writing or educational events | ☐ \| **None** AstraZeneca \| Disease state presentation. Received travel support AstraZeneca is a partner of the SPIROMICS program and partnered in the NHLBI CAPTURE validation study; GSK \| Disease state presentation. Received travel support. GSK is a partner of the SPIROMICS program and partners in the NHLBI CAPTURE validation study; Roche \| Disease state presentation. No honoraria or travel support. | |
| **6** | | Payment for expert testimony | ☐ \| **None** | |
| **7** | | Support for attending meetings and/or travel | ☐ \| **None** | |
| **8** | | Patents planned, issued or pending | ☐ \| **None** | |
| **9** | | Participation on a Data Safety Monitoring Board or Advisory Board | ☒ \| **None** | |
| **10** | | Leadership or fiduciary role in other board, society, committee or advocacy group, paid or unpaid | ☒ \| **None** | |
| **11** | | Stock or stock options | ☒ \| **None** | |
| **12** | | Receipt of equipment, materials, drugs, medical writing, gifts or other services | ☐ \| **None** AstraZeneca \| Writing support; Chiesi \| Writing support; GlaxoSmithKline \| Writing support; Sanofi/Regeneron \| Writing support | |
| **13** | | Other financial or non-financial interests | ☒ \| **None** | |
|  | |  |  | |
| **Please place an “X” next to the following statement to indicate your agreement:** | | | | |
| ☒ | | I certify that I have answered every question and have not altered the wording of any of the questions on this form. | | |
